# Insights of SEDRIC, the Surveillance and Epidemiology of Drug-Resistant Infections Consortium

**DOI:** 10.12688/wellcomeopenres.23494.1

**Published:** 2025-01-13

**Authors:** Nicholas Feasey, Raheelah Ahmad, Elizabeth Ashley, Rifat Atun, Kate S Baker, Francesca Chiari, H. Rogier van Doorn, Alison Holmes, Timothy Jinks, Andrew Jermy, Jyoti Joshi, Souha S Kanj, Matt King, Direk Limmathurotsakul, Janet Midega, Mirfin Mpundu, Jamie Nunn, Iruka N. Okeke, Stuart Reid, Dawn Sievert, Paul Turner, Kamini Walia, Sharon J Peacock

**Affiliations:** 1 Department of Clinical Sciences,, Liverpool School of Tropical Medicine, Liverpool, UK; 2 University of St Andrews School of Medicine, St Andrews, Scotland, UK; 3 NIHR Health Protection Research Unit (HPRU) in Healthcare Associated Infections and Antimicrobial Resistance, Imperial College London, London, UK; 4 Department of Health Services Research and Management, University of London, London, England, UK; 5 Institute of Business and Health Management Dow University of Health Sciences, Karachi, Pakistan; 6 University of Oxford Nuffield Department of Medicine, Oxford, England, UK; 7 Harvard School of Public Health, Boston, Massachusetts, USA; 8 Genetics, University of Cambridge, Cambridge, UK; 9 University of Liverpool Institute of Infection Veterinary and Ecological Sciences, Neston, England, UK; 10 Drug resistant infections Priority Programme, Wellcome Trust, London, UK; 11 Centre for Tropical Medicine and Global Health, University of Oxford, Oxford, UK; 12 Germinate, Germinate Science Consulting Ltd, Suffolk, UK; 13 International Centre for AMR Solutions, Copenhagen, Denmark; 14 Division of Infectious Diseases, Internal Medicine Department, and Center for Infectious Diseases Research, American University of Beirut Medical Center, Beirut, Lebanon; 15 ARCTA solutions Ltd, Hampshire, UK; 16 Mahidol Oxford Tropical Medicine Research Unit, Bangkok, Bangkok, Thailand; 17 ReAct Africa, Lusaka, Zambia; 18 London School of Hygiene & Tropical Medicine, University of London, London, England, UK; 19 Department of Pharmaceutical Microbiology, University of Ibadan, Ibadan, Oyo, Nigeria; 20 The Royal Veterinary College, London, England, UK; 21 Antimicrobial Resistance Coordination and Strategy Unit, National Center for Emerging and Zoonotic Infectious Diseases, US Centers for Disease Control and Prevention, Atanta, Georgia, USA; 22 Cambodia Oxford Medical Research Unit, Siem Reap, Cambodia; 23 Indian Council of Medical Research, New Delhi, Delhi, India; 24 Medicine, University of Cambridge, Cambridge, UK

**Keywords:** Antimicrobial resistance, surveillance

## Abstract

The increasing threat from infection with drug-resistant pathogens is among the most serious public health challenges of our time. Formed by Wellcome in 2018, the Surveillance and Epidemiology of Drug-Resistant Infections Consortium (SEDRIC) is an international think tank whose aim is to inform policy and change the way countries track, share, and analyse data relating to drug-resistant infections, by defining knowledge gaps and identifying barriers to the delivery of global surveillance. SEDRIC delivers its aims through discussions and analyses by world-leading scientists that result in recommendations and advocacy to Wellcome and others. As a result, SEDRIC has made key contributions in furthering global and national actions. Here, we look back at the work of the consortium between 2018-2024, highlighting notable successes. We provide specific examples where technical analyses and recommendations have helped to inform policy and funding priorities that will have real-world impact on the surveillance and epidemiology of infections with drug-resistant pathogens.

## Plain language summary

The increasing threat from infections that cannot be treated with medicines, so called drug resistant infections, is among the most serious public health challenges of our time. Formed by Wellcome in 2018, the Surveillance and Epidemiology of Drug-Resistant Infections Consortium (SEDRIC) is an international think tank whose aim is to inform policy and change the way countries track, share, and analyse data relating to drug-resistant infections, by defining knowledge gaps and identifying barriers to the delivery of global surveillance. SEDRIC delivers its aims through discussions and analyses by world-leading scientists that result in recommendations and advocacy to Wellcome and others. As a result, SEDRIC has made key contributions in furthering global and national actions. Here, we look back at the work of the consortium between 2018-2024, highlighting notable successes. We provide specific examples where technical analyses and recommendations have helped to inform policy and funding priorities that will have real-world impact on the surveillance and of infections with pathogens that are becoming difficult or impossible to treat.

## 1. Introduction

Drug-resistant infections are among the most serious public health challenges of our time. Continued access to effective antimicrobial drugs is vital to safeguard the gains made in global health and development since their commercial production began in the 1940s. However, the widespread use and overuse of antimicrobials in humans, animals, and for agriculture has accelerated the evolution and spread of resistance to front-line antimicrobial drugs. Concomitantly, the market to develop new antimicrobials is restricted as these drugs are less profitable over the long run compared to other classes of medications, leading pharmaceutical companies to deescalate or discontinue their antimicrobial research and development programmes. As a result, we face rising levels of antimicrobial resistance (AMR), with a dwindling pipeline of new drugs with which to stem the tide ^1^.

While AMR is a global problem, in terms of mortality, morbidity and socio-economic impacts, it disproportionately affects populations in low- and middle-income countries (LMICs) ^2^. Tackling AMR is therefore a complex, long-term challenge that will require ongoing multi-pronged international responses involving: the economic and technological reinvigoration of the discovery pipeline for new antimicrobial drugs; exploration of alternative treatment modalities; increased use of available vaccines to support prevention efforts the development and implementation of rapid point-of-care diagnostics to inform effective patient management; improved antimicrobial stewardship; better water, sanitation and hygiene; and the strengthening of laboratory detection and infection prevention and control capabilities ^3– 5^.

High quality surveillance is vital to many of these efforts, particularly in LMICs where resources and data are currently sparse. At present, surveillance largely relies on a combination of representative data and the appropriate collection and testing of samples from patients with a suspected infectious disease presenting at a clinic or hospital, and from animals and the environment. The resulting data can be collated to generate a picture and/or benchmark of current infection and AMR trends, and inform policy decisions at local, national, and international levels ^6^. It is often the case in LMIC that data are not representative as specimens are frequently only collected and tested from the most severe illnesses, after failed treatments and that data are confined to isolates rather than being associated with clinical outcome data. Understanding how the patterns of susceptibility and resistance change geographically over time for a range of bug-drug combinations is vital to inform clinical management, antibiotic stewardship, infection prevention and control, and prioritisation in research and development. Furthermore, having clear sight of the true global health, agricultural, environmental and economic burden of both antimicrobial usage and drug-resistant infections will provide policy makers with the information needed to ensure that funding and resources dedicated to meeting the global challenge posed by AMR are appropriate to the scale of the problem at hand. Accordingly, strengthening the knowledge and evidence base through surveillance and research was included as one of the key components of the WHO Global Action Plan on Antimicrobial Resistance ^7^.

Despite substantial recent progress in developing global AMR surveillance systems ^8, 9^, the landscape remains fragmented, preventing a holistic view of AMR within and across human health, agricultural, veterinary, and environmental sectors ^10– 12^. In recognition of the need to implement, improve and strengthen surveillance at all levels, in January 2018 Wellcome formed the Surveillance and Epidemiology of Drug-Resistant Infections Consortium (SEDRIC), an international ‘think tank’ committed to transforming the way countries generate, track, share, analyse and use information about the rise and spread of drug-resistant infections ^13^.

SEDRIC was established to define gaps in data and knowledge and identify barriers to the delivery of global surveillance. In particular, the aims of SEDRIC were to provide independent scientific analysis, advice and advocacy that: 1) advance and transform how rates of infection and antimicrobial resistance, burden of disease, information on antimicrobial use, and opportunities for intervention are tracked, shared and analysed; 2) strengthen the availability of information needed to monitor and track risks; and 3) support the translation of surveillance data into interventions, changes in policy and more effective practices ^13^. SEDRICs aims and objectives were developed through a gap analysis and Theory of Change process.

At the time of its launch, SEDRIC was guided by a board made up of eleven experts in human and veterinary health, drug-resistant infections, infection control and antimicrobial stewardship, microbiology, epidemiology, genomics, modelling, implementation and delivery of surveillance, and policy making; supported by a secretariat provided by Wellcome that was responsible for organising board meetings, implementing board recommendations, and running day-to-day operations.

**Figure 1.**
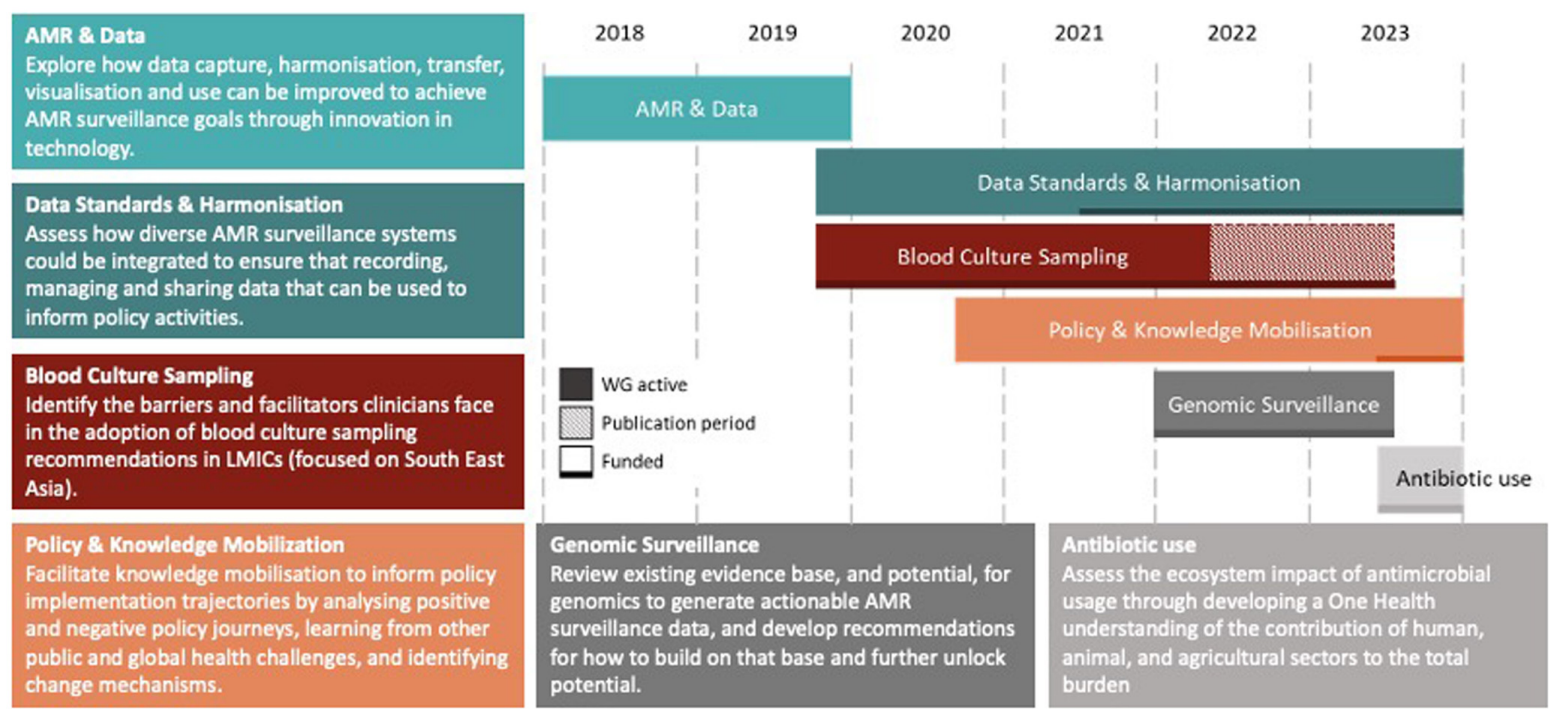
A timeline of SEDRIC Working Groups 2018–2023. Six working groups have been convened and delivered analysis, advice and projects over the first five years of SEDRIC. Periods of research and publication activity, and funding are noted.

The SEDRC board was complemented by a wider network of members contributing to consortium activities by joining annual consortium meetings, developing and writing reviews and opinion articles, as well as participating in, and leading, dedicated working groups focused on gaps in knowledge, data, and research, identified by the SEDRIC board. Since launch, there have been six working groups ( Figure 1). Three of these groups have completed their analyses and have generated multiple outputs, while three remain active.

Through the work of the board, secretariat, membership and working groups, SEDRIC was intended to provide thought leadership and technical expertise on existing surveillance networks and the wider field, in line with the WHO Global Action Plan for AMR objective to strengthen the evidence base through surveillance and research. By doing so, SEDRIC would help to shape the surveillance and epidemiology strategy for Wellcome’s Drug-Resistant Infections programme, which ran over the period 2017–2021.

In this report, we review the work of SEDRIC from 2018–2024 to compare initial expectations for the consortium against what has been achieved, highlighting notable successes and identifying areas of need for strengthening of surveillance and epidemiological understanding of AMR. Using specific examples, we examine how SEDRIC has undertaken technical analyses, generated high quality advice and products, and advocated for discrete policy and funding priorities that are already resulting in real-world impact on the surveillance and epidemiology of drug-resistant infections.

## 2. Thought leadership in AMR surveillance

A major part of SEDRIC’s goal was to become a high-level independent advisory body to guide Wellcome, and other national and international bodies, in better understanding the gaps in data and knowledge, identifying barriers to the delivery of global AMR surveillance, and exploring opportunities for solutions ^13^. Below, we describe some examples.

### 2.1 Alternative data sources

Identifying under-utilised existing sources of surveillance data that can inform our understanding of the epidemiology of AMR was identified by the SEDRIC board as a key area for attention. This followed an analysis commissioned by the Fleming Fund ^14^ which characterised the LMIC AMR surveillance landscape, finding seventy-two supranational surveillance networks operating between 2000–2017, led by governments or the WHO (n=26), academics (n=24), or pharmaceutical companies (n=22) ^10^. The fragmentation and heterogeneity observed, with a lack of coordination, harmonisation and data sharing between the various local, national, and international initiatives reflects the pattern found in high income countries (HICs) in Europe ^15^. SEDRIC members explored how data from pharmaceutical, academic, and private laboratories could be integrated to help fill in the gaps until the capacity to undertake standardised surveillance as routine across all LMICs has been established ^16^. Multiple barriers were identified, including: i) restrictions on data access, often owing to delayed release until publication of research findings, legal and ethical hurdles to making certain types of data public, or the generation of data only intended for internal use within pharmaceutical companies; ii) lack of harmonisation in data collection between networks; iii) lack of user-friendly tools for data collection and deposition; and iv) lack of a framework agreement to protect those generating data against negative impacts of sharing data early so that it can be used by others. It was also noted that the WHO Global AMR Surveillance System (GLASS), currently the most prominent global surveillance initiative for bacterial pathogens, cannot accept information generated by research or pharmaceutical industry activities ^8, 17^. The working group identified key actions required to harness these data, including: i) a mapping exercise to evaluate the quality and utility of existing data and how it can be strengthened to inform surveillance; ii) identifying mechanisms to incentivise greater data sharing; iii) creating channels to support the use of data from pharma, academic and private laboratories by national and international surveillance programmes; and iv) driving innovation in data capture, analysis and sharing.

This analysis, and the advice generated by SEDRIC members directly informed the design of several projects funded by Wellcome, including ACORN (A Clinically Oriented antimicrobial Resistance surveillance Network) ^18^. It also led to the establishment of a working group (external to SEDRIC) with representatives from Wellcome, Public Health England (replaced in 2021 by UK Health Security Agency and Office for Health Improvement and Disparities) and University College London which explored how best to create an open access platform to share industry surveillance data, which is now hosted by Vivli ^19^. This platform further enabled the creation of Data Re-Use Prize programmes, where researchers were able to combine various datasets to find new ways to do surveillance ^20^.

### 2.2 Global burden of disease for AMR

Without an accurate understanding of disease burden owing to drug-resistant infections, it is impossible to meaningfully estimate the scale of the problem and therefore the resources that need to be devoted to meet the challenge. Understanding whether a patient dies ‘from’ or ‘with’ a drug-resistant infection is vital for determining the true global burden of disease owing to AMR. However, studies that have attempted to estimate the global burden of disease owing to AMR have been hampered by a severe lack of data and have used multiple approaches, each with its own set of pros and cons ^21^. A SEDRIC analysis found that owing to differences in methodology, and the assumptions and data used, existing estimates of global AMR burden cannot be compared ^22^. 

Healthcare systems worldwide use International Classification of Diseases (ICD) codes to document medical conditions on patient records ^23^. While death often results from a complex interplay of conditions, it is standard for only a single cause of death to be recorded. Relying on ICD means that deaths are attributed to the condition that led to hospitalisation, even if death was a direct result of a hospital-acquired infection. As such, ICD-based surveillance is likely to substantially underestimate the true global burden of disease directly attributable to drug-resistant infections ^24^. An alternative is to use all-cause mortality, which includes any death for which a drug-resistant infection is involved, even if it is not directly attributable, which risks overestimating disease burden owing to AMR (e.g. Ref 25). Another approach is to determine attributable mortality, which uses the counterfactual assumption that death would not have happened if the infection had not occurred or was not drug-resistant, which can be determined by comparing to outcomes in uninfected patients or in patients with drug-susceptible infections. Attributable mortality approaches can help to avoid overestimates/underestimates but are affected by data availability and an over-reliance on data from HICs to infer estimates in LMICs ^21^.

Concluding that the analytical frameworks used in previous studies were inadequate and that new inclusive approaches to estimate deaths caused by AMR infection were needed, SEDRIC advised that the creation of a systematic clinical dataset of substantial breadth and quality was necessary to support the accurate assessment of AMR burden ^22^. These findings were presented at a workshop organised by Wellcome (Measuring the global burden of antimicrobial resistance: Exploring methods, models, and best practices; 5th July 2019) attended by 30 global experts including researchers leading The Global Research on Antimicrobial Resistance (GRAM) Project ^26^. GRAM have subsequently provided the most recent and comprehensive assessment of global disease burden attributable to AMR, which estimated that in 2019 there were 4.95 million (3.62–6.57) deaths associated with, and 1.27 million (95% UI 0.911–1.71) deaths directly attributable to bacterial AMR ^2^; several members of the SEDRIC board co-authored this seminal work.

### 2.3 Policy and Knowledge Mobilisation

The Policy and Knowledge Mobilisation working group was convened to accelerate learning and facilitate knowledge mobilisation to inform policy implementation trajectories in countries and regions. The activities of this group include optimising the use of research-generated knowledge, and encompass dissemination, knowledge transfer (where emphasis is on knowledge-push), and knowledge exchange, with an emphasis on action. The specific objectives of the working group are to: i) conduct analysis of policy journeys of positive and negative outlier example countries; ii) understand the relevance to AMR, of success in other public health and global health challenges; iii) identify generalisable mechanisms which lead to change; and iv) share this learning with specialists in AMR as well as wider stakeholders in public health and global health policy development and implementation and evaluation. The working group is mindful to mitigate the risk of ‘talking to ourselves’ as field experts, to self-assess the ‘knowledge management maturity’ ^27^ of SEDRIC, ensure links with other global think-tanks to avoid duplication of effort, and to provide evidence from diverse geographical and resourced/under-resourced settings to help understand ways forward for implementation. The working group has hosted a series of webinars and emphasised experiences of early career practitioners and researchers. This included an event that was among the earliest seeking to capture experiences and share learnings on how COVID-19 impacted AMR surveillance, which was followed by an analysis of the structures and processes which supported knowledge mobilisation during the pandemic ^28^.

In August 2023, the group initiated an ambitious project to qualitatively map AMR policy interventions that have been adopted by countries. The analytic approach extends earlier cross-country comparative analysis of infection control interventions developed by working group members ^29, 30^ and their collaborators ^31^. The aim is to provide country level decision makers with a comprehensive suite of policy options and a method for tracking policy implementation so that evaluative work is possible alongside AMR burden estimates. The study protocol is available ^32^, while the first 17 case country analyses progresses, so that there is no delay in allowing countries to start the analysis or to nominate their country to the working group to support analysis. Results of the work will be available via an interactive dashboard with links to the GRAM study embedded. Collaboration has extended to Fleming Fund Policy Fellows through training and involvement in country analysis. The outputs align closely to the evidence required to take forward recommendations from the United Nations high level meeting 2024.

## 3. Patient-centred surveillance

Most existing approaches to AMR research and surveillance were designed and developed with high-income settings in mind. However, few data are available on how best to prioritize efforts in settings where human and financial resources are limited, and which often have distinct competing health needs ^33^. SEDRIC sought to address this knowledge gap and understand what a patient-centred approach, which places the need for improved surveillance secondary to meeting the immediate healthcare needs of individuals with drug-resistant infections, might require in low-income settings.

### 3.1 Priority setting for patient-centred surveillance

The first working group to be established focussed on AMR and Data ( Figure 1) and was given the brief of defining short- to medium-term priorities for AMR research and surveillance to benefit human health in LMICs. The working group sought to rank the competing health priorities by developing a transparent priority-setting process (PSP) driven by stakeholders working in LMICs ^34^ using a multilingual online survey to generate a list of uncertainties related to AMR surveillance and human health in LMICs. The survey was disseminated through social media and informal networks and generated 1076 questions from 445 respondents, 80% of which were practicing clinicians or microbiologists involved in patient care in LMICs. Responses were organised into themes, a ranked shortlist of questions generated, and by consensus the steering group identified the following three questions to be recommended as priorities for further research to the SEDRIC board:

1. Which infection prevention and control (IPC) interventions should be prioritised in LMICs, considering the context (overcrowding, no isolation facilities, poor infrastructure, low availability of PPE, water, and sanitation issues) and limited financial resources?
2. What is the role of improved information patient management systems, including electronic prescribing, to tackle AMR?
3. How can we bring about sustainable behaviour change among doctors and other health care professionals concerning managing infections and prescribing antibiotics?

**Figure 2.**
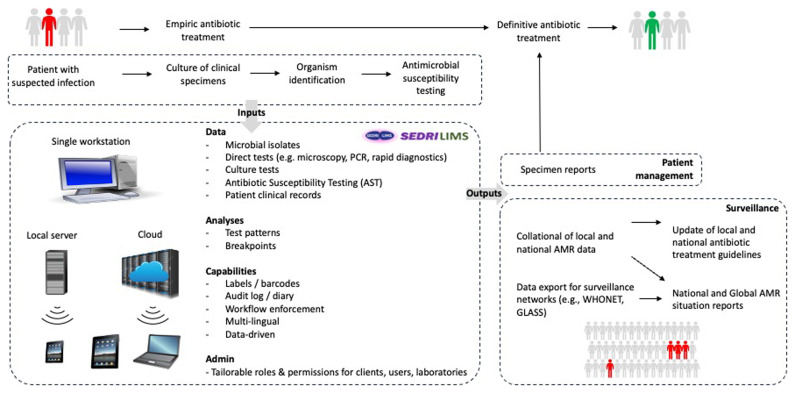
An overview of SEDRI-LIMS.

The resulting study was widely read across Wellcome and informed the formation of two new SEDRIC working groups: i) the Data Standards & Harmonisation working group, to advocate for and lead the development of an open-source laboratory information management system (LIMS) ( Figure 2; Section 3.2); and ii) the Blood Culture Sampling working group, to identify the barriers and facilitators clinicians face in the adoption of blood culture sampling recommendations in South East Asia ( Table 1; Section 4.1).

**Table 1.** SEDRIC impacts from gap identification to outcome.

Gap	Analysis	Advice	Advocacy	Output	Impact
Alternative data sources for AMR surveillance	• Building on Fleming fund- sponsored review of LMIC surveillance networks • Identify potential additional sources of data for use in short- to medium- term • Description of barriers to using data	• Mapping exercise to evaluate the quality and utility of existing data • Identify mechanisms to incentivise greater data sharing • Create channels for data linking pharma, academic and private labs with national and international surveillance programmes • Prioritise innovation in data capture, analysis and sharing.	• Publication of final report • Discussion with Wellcome and other funders	Ashley *et al*., 2018 (Ref 10)	Impact Fed into thinking behind: • Wellcome data re-use prize • Vivli AMR programme
View of global disease burden for AMR	• Consideration of the pros and cons of different analytical frameworks uses to estimate burden of disease attributable to AMR.	• Create a systematic clinical dataset of substantial breadth and quality to support the accurate assessment of burden • In the absence of adopting a universally agreed best practice, disease burden researchers to consider and report pros and cons of methodology being adopted.	• Publication of final report • Discussion with Wellcome and other funders • Findings presented at workshop on Measuring the global burden of antimicrobial resistance hosted by Wellcome	Limmathurotsakul, 2019 (Ref 22)	
Patient- centred surveillance priority setting	• Priority setting process surveying 445 clinicians and microbiologists • Collating and ranking 1076 questions • Identification of three priority research questions	• Identify most suitable IPC measures for LMICs • Develop improved LIMS • Incentivise behaviour change among healthcare professionals managing infections	• Continue ongoing research into IPCs • Fund development of an open-source LIMS suitable for use in LMICs • Fund study of barriers to behaviours change	Ashley *et al*., 2020 (Ref 34) Turner *et al*., 2021 (Ref 35)	• Formation of Data Standards and Harmonisation Working Group • Funding of SEDRI LIMS development by SEDRIC and ARCTA • Formation of Blood Culture Sampling Working Group
Genomic AMR surveillance	Workshop series with 97 world-leading experts in genomic AMR surveillance considering: • Hospitals • Public health and international networks; • One Health interfaces • Innovations in non-isolate-based genomics • Followed by • Landscape analysis • Expert consensus building • Community survey	Development of series of 9 recommendations relating to: • Defining a use framework for genomics in AMR surveillance • Building capacity • Training • Harmonisation and developing standards • Equitable data sharing and governance • Stakeholder relationships • Funding models • Investing in innovation • Integrating environmental surveillance • Publication of final report • Discussion with Wellcome and other funders	• Publication of executive summary • Publication of an overview and four workshop reports • Discussion with Wellcome and Bill & Melinda Gates Foundation • Presentation of findings at the Gates Grand Challenges, ESCMID IMMEM XIII, and Applied Bioinformatics and Public Health Microbiology conferences	Baker *et al*., 2023 (Ref 37) Jauneikaite *et al*., 2023 (Ref 38) Baker *et al*., 2023 (Ref 39) Muloi *et al*., 2023 (Ref 40) Wheeler *et al*., 2023 (ref 41)	

### 3.2 Development of a laboratory information management system

Clinical microbiology laboratories in LMICs often rely on paper records, with little use of electronic data. While not ideal, at the level of individual patients, paper records can be sufficient to support acceptable levels of healthcare provision. Lack of electronic data, however, is problematic for strengthening of local policies (i.e. in antimicrobial stewardship) and for supranational surveillance programmes such as WHO GLASS ^8^.

Where open-source LIMS are available for use in LMICs, they have not been designed to enable detailed recording and easy extraction of consistent microbiological and drug resistance data ^35^. In the absence of a comprehensive LIMS, there is an inability to use diagnostic information for patient care or to inform facility level IPC measures. SEDRIC advocated for, and subsequently steered the development of an open-source LIMS to provide specimen management, bench workflow, result reporting, automated and manual analysis of AMR data and to interface with local hospital systems, as well as national and international surveillance initiatives including WHONET and GLASS ( Figure 2). Arcta, a professional software house specialising in systems for healthcare management ^36^, were contracted by Wellcome to develop a proof-of-concept pilot over the course of 18 months, followed by 3 months of pilot testing. ARCTA used an agile approach to develop the LIMS, building the software piece by piece, and engaging with stakeholders globally to demonstrate functionality and then improving iteratively as the pilot grew.

The high level of engagement seen from those present in steering group meetings and product demonstrations highlighted the global need for a LIMS designed with clinical microbiology data in mind.

Following the successful pilot, Wellcome funded a second phase of development and real world testing over three further years to: i) include integration with a range of clinical laboratory testing machines; ii) add functionality to ensure that antimicrobial susceptibility testing data are robust and easy for sites to update; iii) devise expert rule systems so that the LIMS could interpret complex AMR patterns and give appropriate comments on treatment; iv) build in other laboratory disciplines (e.g. haematology and biochemistry); and iv) undertake early adoption testing at a larger number of locations. Wellcome is currently exploring the options to best deploy the mature product to laboratories globally, once the further development work is completed in 2025.

The development of a LIMS as a product is one of the major outcomes of the work of SEDRIC and reflects the aims of the consortium to leverage expert analysis to identify a gap in knowledge or surveillance data, provide valuable expert advice on how best to address the gap, and then advocate for funding to address a critical need in global AMR surveillance, outside a standard research funding framework. While the involvement of SEDRIC in the project is nearing completion, its central role in the genesis of the project is recognised in the choice of ‘SEDRI LIMS’ as the name for the mature product that will be deployed over the coming years and, will have a major impact on strengthening AMR surveillance and epidemiology.

## 4. Surveillance technology and low-income country settings

Relying on culturing and antibiotic susceptibility testing of clinical isolates is slow and labour-intensive, limiting the potential benefits for infection management. Advances in a wide range of technologies promise to revolutionize the treatment, surveillance, and epidemiology of drug-resistant infections, from new approaches to culturing, point-of-care pathogen identification and resistance detection, genomic sequencing, through to machine learning and artificial intelligence. While, supporting laboratories to ‘leapfrog’ conventional microbiology approaches to adopt high tech solutions could help to close resistance surveillance gaps, there are numerous pitfalls and realising the potential benefits of these technologies in the short to medium term may be prohibitively expensive ^42^. Furthermore, in many lower-income country settings, there are often low-tech options that have yet to be adopted as standard that will likely deliver more immediate benefits to patients. SEDRIC undertook analyses to identify gaps and barriers to adoption of low tech (blood culture sampling) and high tech (genomics) solutions in LMICs.

### 4.1 Blood culture sampling working group

Blood culture sampling is a critically important diagnostic tool in the management of sepsis that can help to inform antibiotic treatment decisions and improve patient outcomes, as well as providing useful information for surveillance efforts. Use of blood culture sampling varies widely, with barriers to uptake including lack of guidelines, training, lack of microbiology laboratory infrastructure and physician attitude to blood culture ^43^. This is a critical knowledge gap given that the UK government has invested over £500M in strengthening blood culture capacity in LMICs via the Fleming Fund. To better understand these barriers, and in response to the third priority question identified by the PSP ( Section 3.1), the SEDRIC board formed the Blood Culture Sampling working group, to identify barriers and enablers to blood culture sampling in three LMICs in Southeast Asia.

The working group surveyed 1070 medical doctors and 238 final year medical students from Indonesia, Thailand, and Vietnam, finding that likelihood of use of blood culture sampling in a patient presenting with community-acquired sepsis varied from 30% to 90% across the three countries ^43^. Heterogenous barriers and enablers to blood culture sampling were identified, including: prioritisation of blood culture; perception of roles in initiation blood culture; perception of the benefits of blood culture; awareness of and desire to follow institutional guidelines; potential consequences that discourage blood culture sampling; cost and perceived cost-effectiveness of blood culture; and regulation on cost reimbursement.

Policy makers and researchers advocating for the application of high-tech solutions in LMICs often miss that even in high income settings, conventional approaches such as blood culture sampling continue to be routinely performed. Having a cultured isolate of an infecting pathogen enables identification, simple, accurate antibiotic susceptibility testing and whole genome sequencing. As genomic technologies develop, routine surveillance without culture may become more feasible. However, it remains unrealistic in the short- to medium-term in both high income and LMIC settings, and so understanding the spectrum of behavioural influences that affect adoption of existing, validated technology like blood culture sampling will be vital in widening the use of diagnostic tests with the potential to save lives today. The findings of this analysis have been noted by the Fleming Fund, which is placing patient centred surveillance at the heart of its second phase and is engaging with SEDRIC in support of this aim.

### 4.2 Genomic surveillance working group

**Figure 3.**
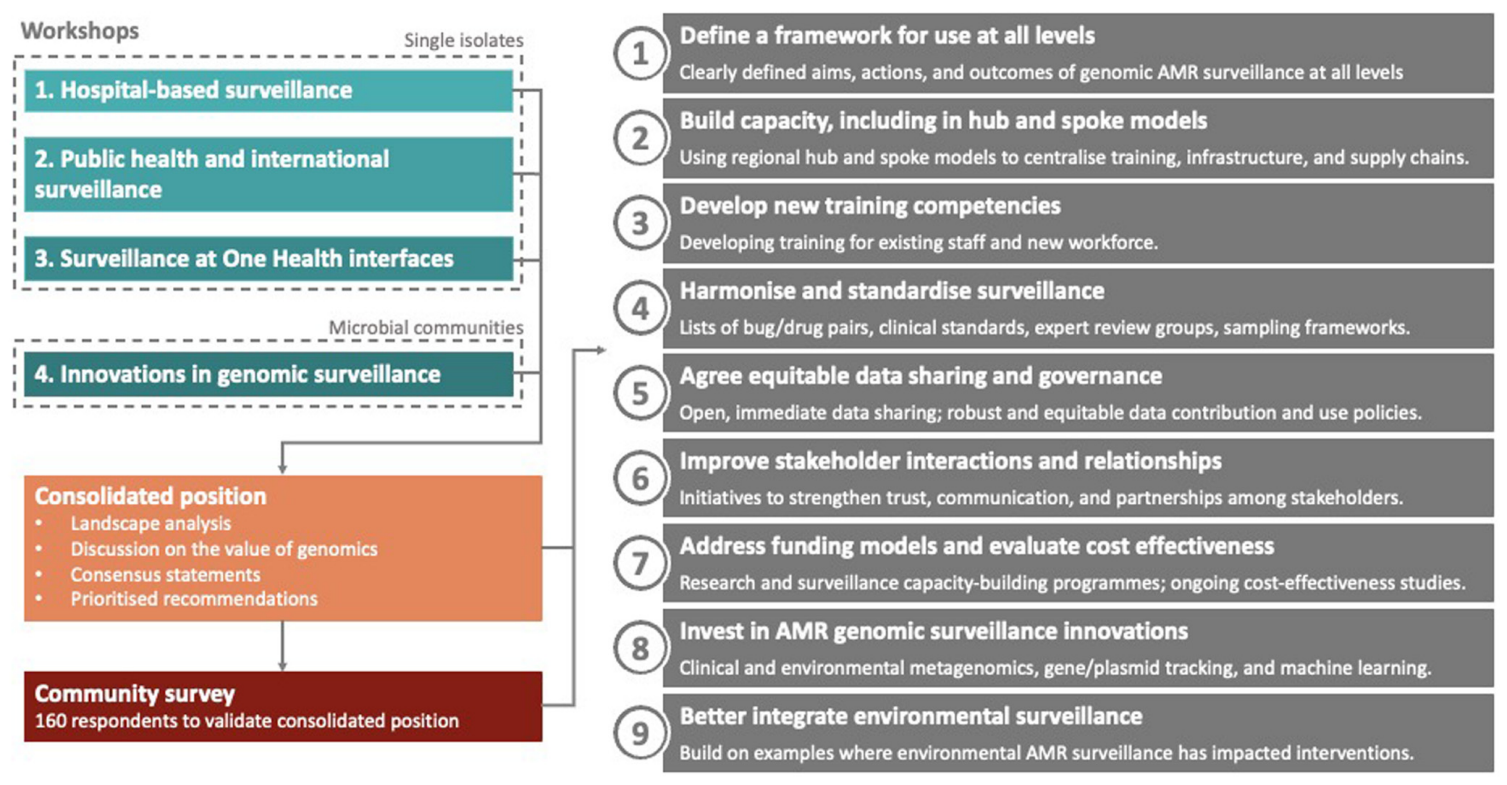
Evidence base review process and recommendations from Genomics Surveillance for AMR Working Group.

Recognizing the potential that genomics has to contribute to surveillance barriers ^36^ and having seen the potential of genomic surveillance realised at unprecedented scale to track SARS-CoV-2 transmission and monitor evolution during the pandemic ^44^, SEDRIC convened a working group to review Genomic Surveillance for AMR. The group held a series of workshops, attended by ninety-eight world-leading experts from across the AMR and pathogen genomics fields ( Figure 3). The first three workshops were focused on situations where genome sequencing of individual pathogen isolates can be used for: i) hospital-based AMR surveillance; ii) public health and international AMR surveillance; and iii) AMR surveillance at One Health interfaces. A fourth workshop focused on innovations in genomics relevant for non-isolate focused AMR surveillance (including clinical metagenomics, environmental metagenomics, gene and plasmid-based tracking, and machine learning). Collectively, these workshops represented a thorough landscape analysis and consensus building exercise on existing and future opportunities for genomic AMR surveillance. A community survey of a further 160 people working in the field was also undertaken, finding broad agreement with the expert consensus. Finally, the working group prioritised nine recommendations for realising the potential of genomics in these areas.

Genomic approaches were considered to offer many potential advantages over other approaches to AMR surveillance including: i) finely resolved tracking of drug-resistant pathogens at the individual strain level; ii) electronic data allowing automation of sharing, storage, quality assurance and analyses; iii) assessment of genotypic resistance to multiple classes of antimicrobials in parallel, and other relevant phenotypes (e.g. virulence, serotypes); iv) assessment of evolutionary history of resistant lineages; v) determination of genetic basis of resistance allowing links between outbreaks linkage to be identified and the prediction of complex phenotypes and the capacity for AMR spread; and vi) ability to pivot the established capacity for new microbial threats as part of pandemic preparedness efforts.

Applications for genomic AMR surveillance differed by domain and in level of maturity. In hospital settings, genomics can be used for outbreak detection and to inform IPC policies. As genome sequencing and analysis becomes increasingly routine in reference laboratories, it opens the door to pathogen genomics to inform clinical decision making for individual patients in the future. For public health, genomics is already used to monitor for emerging threats and in designing and assessing the efficacy of interventions, for example by informing vaccine formulations. At One Health interfaces genomic technologies are already commonplace for surveillance of foodborne diseases in some regions ^45, 46^, moreover further opportunities for their use as part of risk assessment frameworks, and for environmental monitoring were identified.

The working group identified common barriers to adoption of genomic AMR surveillance which included: i) a lack of resources and political will; ii) the lack of a common framework with clearly identified use cases supported by robust cost effectiveness studies; iii) and insufficient skilled workforce and training infrastructure, particularly around bioinformatics; iv) limited epidemiological surveillance or microbiology infrastructure; v) inadequate supply chains and pricing structures; vi) and a lack of effective stakeholder engagement and cooperation to define common goals and harmonise surveillance and data governance ^37– 41^. Its work inspired the formation of the UK transdisciplinary network on AMR genomics ( https://www.targetamr.org.uk/)

## 5. Underexplored gaps in AMR surveillance and epidemiology

Despite the many advances that have been made in approaches to AMR surveillance in the last few years, significant gaps remain. The challenges associated with AMR surveillance in a One Health context, which seeks to integrate and optimize the health of people, animals, and ecosystems remains underexplored. While One Health was specifically considered within the work of the Genomics Surveillance for AMR working group, there are many challenges beyond the application of genomics that need to be urgently addressed. For example, metrics used to measure human and animal antibiotic usage are incompatible, which limits our ability to track antibiotic uses and consumption globally. Creating a globally endorsed metric that measures antibiotic consumption across the One Health continuum (and critically, quantitatively links this to the emergence of AMR) would be a notable achievement, although would require considerable investment to support widespread adoption. Also, within the One Health context, monitoring wastewater to inform disease surveillance programmes is becoming increasingly commonplace, particularly in urban populations ^47^. Linking wastewater surveillance (WWS) with human surveillance to quantify the impact that resistance determinants and antibiotic residues found in the environment have on human health is another underexplored area, however the signal from WWS is challenging to interpret due to the complex interactions of microorganisms in the environment.

Neonatal sepsis, caused by both drug-susceptible and drug-resistant infections remain a substantial cause of mortality worldwide ^48^. There remain many gaps in knowledge and available data for neonatal sepsis. There is also a potential role for surveillance data to strengthen the role and impact of vaccines against AMR. For example, the Global Pneumococcal Sequencing (GPS) project demonstrated how circulating populations of *Streptococcus pneumoniae* adapt to vaccine introduction by expanding existing lineages to fill the niche vacated by vaccine susceptible strains. Insights from GPS have been used to inform which serotypes to include in updated pneumococcal conjugate vaccines ^49^. Consideration of how lessons from GPS, and other such projects, could be applied to inform future vaccination programmes against a wider range of drug-resistant infections is urgently needed, and maternally administered vaccines to tackle neonatal sepsis is one key example ^50^.

Another gap that warrants greater attention is in linking surveillance data to actions in the clinic and quality of health care in countries in general. At present, there are no consistent protocols for how to use an institutional or national antibiogram to inform changes in treatment guidelines, especially given the lack of linked clinical metadata and the wide variability in the availability and quality of antibiotic susceptibility testing data. If specimens are not collected for testing and clinicians do not trust the lab results, or do not want to use laboratory testing owing to the costs involved, then there are no accurate infection and resistance data to appropriately inform patient treatment, infection prevention and control, and surveillance. Developing toolkits to analyse available information, identify useful signals and provide useful advice to clinicians would be of substantial benefit in the management of patients with drug-resistant infections. In the future, these will benefit from leveraging the potential of machine learning. This will not, however, address the cost for these approaches, nor who pays in LMIC; AMR surveillance requires policy and legislative support to successfully implement improvements.

Beyond the clinic, there remain major challenges to overcome in linking surveillance data to population and public health level metrics, which prevents a comprehensive understanding of the drivers and impacts of drug-resistant infections. This is compounded by barriers to cross-country collaboration for surveillance and data sharing. Advocating for simplified data sharing policies and protocols and greater engagement with national health institutions, particularly in LMICs, as well as international organisations could help to reinforce the value of surveillance for improved healthcare systems and health care delivery into action. This will help to enable the translation of AMR surveillance data into contextually relevant solutions for local implementation, such as investment in human resources outside of microbiology laboratories.

As important a challenge as bacterial AMR poses, there is a wide range of drug-resistant and susceptible pathogens for which existing surveillance data and epidemiological insights are limited or non-existent. The emergence and spread of the multidrug-resistant *Candida auris* is but one example. Whether caused by bacterial, viral, fungal, or protozoan pathogens, there is normally a broad overlap in the individuals and communities that are affected by different communicable diseases. Furthermore, many knowledge gaps, and technical or logistical challenges associated with diagnosing, treating, and sharing data are common to surveillance efforts for many infectious diseases.

## Concluding remarks

Antimicrobial resistance (AMR) is a critical global public health challenge, underscored by the 2024 United Nations General Assembly High-Level Meeting on AMR ^51^. The resulting political declaration set ambitious goals, which included targets a 10% reduction in global AMR-related deaths by 2030 and a decrease in antibiotic use. Achieving these goals hinges on the generation of robust AMR surveillance data, and this requires sustained investment, coordinated global efforts, and the adoption of both existing/effective tools and cutting-edge technologies. SEDRIC has played a pivotal role in shaping the understanding of the AMR surveillance requirements reflected in the UNGA declaration, which will drive progress towards these essential targets.

## Ethics and consent

Ethical approval and consent were not required

## Disclaimer

The views expressed in this article are those of the authors. Publication in Wellcome Open Research does not imply endorsement by Wellcome

## Data Availability

No data are associated with this article
