# Supplementary material for: Insights of SEDRIC, the Surveillance and Epidemiology of Drug-Resistant Infections Consortium
Source: Wellcome Open Res. 2025 Jan 13;10:5. [Version 1] doi: 10.12688/wellcomeopenres.23494.1 (PMC11885901; doi:10.12688/wellcomeopenres.23494.1)
Supplement: Peer review history

# Reviewer report: Reviewer response for version 1

**DOI:** 10.21956/wellcomeopenres.25914.r120130

**Published:** 2025-03-11

**Authors:** Francis Kakooza

**Affiliations:** 1 Makerere University Refugee Law Project, Kampala, Central Region, Uganda

Well written. More emphasis on linking SEDRIC efforts to the GLASS implementation and reporting. This should include details on quality assured microbiology testing capacities (pathogen priorities and AST capacities). In addition, data quality assurance for all the surveillance data and information sharing across the consortium. Finally, it's a good scientific report for the global AMR team

Does the article adequately reference differing views and opinions?

Yes

Are all factual statements correct, and are statements and arguments made adequately supported by citations?

Yes

Is the Open Letter written in accessible language?

Yes

Where applicable, are recommendations and next steps explained clearly for others to follow?

Yes

Is the rationale for the Open Letter provided in sufficient detail?

Yes

Reviewer Expertise:

Global Health Security; AMR Surveillance; Microbiology; Public Health Emergencies

I confirm that I have read this submission and believe that I have an appropriate level of expertise to confirm that it is of an acceptable scientific standard.

# Reviewer report: Reviewer response for version 1

**DOI:** 10.21956/wellcomeopenres.25914.r117204

**Published:** 2025-03-06

**Authors:** Marcelo Pillonetto

**Affiliations:** 1 Pontifícia Universidade Católica Do Paraná, Curitiba, Paraná, Brazil; 2 Molecular Bacteriology, State Reference Laboratory of Public Health of Paraná, Curitiba, PARANÁ, Brazil

**Summary of the Article ** 

 Thanks for the opportunity to review such an outstanding manuscript, by Feasey et al. 

 The article titled "Insights of SEDRIC, the Surveillance and Epidemiology of Drug-Resistant Infections Consortium" focuses on the Surveillance and Epidemiology of Drug-Resistant Infections Consortium (SEDRIC), an international think tank established by Wellcome in 2018. It positions SEDRIC as a pivotal initiative for addressing the increasing threat posed by infections with drug-resistant pathogens, one of the most critical public health challenges globally. Its primary objectives include identifying barriers to effective antimicrobial resistance (AMR) surveillance, defining knowledge gaps, and informing policies through global advocacy and technical analyses. By exploring its achievements and contributions from 2018 to 2024, the article outlines SEDRIC's efforts to improve how countries track, share, and analyze data related to drug-resistant infections. Furthermore, it emphasizes the crucial role of global collaboration and advanced tools, such as professional surveillance software, in achieving meaningful progress in the fight against AMR. The article is well-written and provides valuable insights into SEDRIC's work and its broader implications for antimicrobial surveillance policy. The emphasis on global data-sharing frameworks and the acknowledgment of persistent challenges give depth to its narrative. However, the article could be improved in some areas to enhance its comprehensiveness and inclusivity.  

 Strengths  

- The article successfully communicates the scale of the AMR challenge and articulates why collaborative frameworks like SEDRIC are essential.  

- It bridges technical outputs with actionable policy recommendations, facilitating informed decision-making.  

- The mention of technical solutions, such as developing tools like Arcta, demonstrates the need for tailored innovations to improve AMR surveillance capabilities globally.  

**Points for Improvement**

 While the article is insightful, the following areas need further attention: 

- Updated the numbers of deaths on the final sentence of item 2.3 referencing newer global literature on AMR Bruden, such as "Global Burden of Bacterial Antimicrobial Resistance, 1990–2021" 

( https://doi.org/10.1016/S0140-6736(24)01867-1 )

  

- The article lacks sufficient representation of LMICs, particularly Latin America, in discussing SEDRIC's contributions. Countries such as Argentina, Brazil, Chile, and Colombia have generated significant epidemiological surveillance data, including many studies cited here. Incorporating their input into the dialogue would strengthen SEDRIC's legitimacy as a global initiative. 

- Recommended Action: Collaborate in the future with or highlight findings from these regions to ensure inclusivity and represent their unique challenges and contributions to AMR surveillance. For example, BR-GLASS provides critical data on resistance mechanisms and real-world surveillance in Brazil, serving as a regional model.  

( https://doi.org/10.3389/fpubh.2020.575536) 

  

- The authors focus on whole-genome surveillance as one of the two additional working groups for SEDRIC, monitoring AMR progression, and blood sample collection. While undoubtedly a valuable tool, it overlooks more affordable and practical methodologies, such as PCR-based surveillance systems. PCR offers a reliable and cost-effective alternative for LMICs with limited funding and infrastructure, and its absence from the discussion restricts the article's relevance for these regions.  

- Recommended Action: Include the discussion about the utility of PCR methodologies in AMR surveillance programs and highlight how combining them with genomic approaches can enhance cost-effectiveness and feasibility, especially in LMIC contexts.  

- The manuscript's emphasis on LIMS is commendable, as the absence of comprehensive systems limits operational efficacy and comparative research. Developing integrated LIMS tailored to LMIC needs should be prioritized to streamline diagnostics and inform facility-level infection prevention and control (IPC) measures.  

- The article highlights the role of organizations such as Arcta, which design healthcare management systems tailored to AMR surveillance needs. This is a promising step, but its integration must prioritize accessible and scalable solutions. A roadmap for collaboration between software developers and public health authorities could further enhance the deployment of such tools.  

- Addressing the resistance shifts observed during COVID-19, citing numbers and examples could provide more comprehensive and actionable insights for countries navigating post-pandemic AMR dynamics.  

Final Comments  

 The article is an essential contribution to the discourse on global AMR surveillance. However, its effectiveness could be improved through enhanced inclusivity, methodological diversity, and actionable recommendations tailored to LMICs. Addressing these points will make the article more scientifically sound and strengthen its practical relevance for policymakers and health professionals worldwide.  

 Recommended Revisions to Improve Scientific Soundness  

1. Representation and data from LMICs, particularly Latin America, should be incorporated to ensure inclusivity.  
2. Expand the discussion on viable economic surveillance methodologies like PCR, making the insights more applicable to LMICs.  
3. Address challenges in data comparability and standardization, providing specific solutions for global alignment.  
4. Integrate insights from studies on the impact of the COVID-19 pandemic on AMR burden to offer timely recommendations.  

1. Thoughts on how to impact policy makers and their willingness to fight AMR, which is a significant concern for LMIC. 

Although we consider the manuscript very robust, with these revisions, the article will achieve a broader perspective on the global AMR fight, supporting its position as a guiding reference for researchers, policymakers, and public health advocates.

Does the article adequately reference differing views and opinions?

Partly

Are all factual statements correct, and are statements and arguments made adequately supported by citations?

Yes

Is the Open Letter written in accessible language?

Yes

Where applicable, are recommendations and next steps explained clearly for others to follow?

Yes

Is the rationale for the Open Letter provided in sufficient detail?

Yes

Reviewer Expertise:

Clinical Microbiology, Public Health Surveillance, AMR molecular detection, and AMR surveillance and policies

I confirm that I have read this submission and believe that I have an appropriate level of expertise to confirm that it is of an acceptable scientific standard.

## References

[1] : Global burden of bacterial antimicrobial resistance 1990-2021: a systematic analysis with forecasts to 2050. *Lancet* .2024;404(10459) : 10.1016/S0140-6736(24)01867-1 1199-1226 10.1016/S0140-6736(24)01867-1 39299261 PMC11718157

[2] : The Experience of Implementing a National Antimicrobial Resistance Surveillance System in Brazil. *Front Public Health* .2020;8: 10.3389/fpubh.2020.575536 575536 10.3389/fpubh.2020.575536 33520909 PMC7841397

[3] : Update on the epidemiology of carbapenemases in Latin America and the Caribbean. *Expert Rev Anti Infect Ther* .2021;19(2) : 10.1080/14787210.2020.1813023 197-213 10.1080/14787210.2020.1813023 32813566

[4] : A 7-Year Brazilian National Perspective on Plasmid-Mediated Carbapenem Resistance in Enterobacterales, Pseudomonas aeruginosa, and Acinetobacter baumannii Complex and the Impact of the Coronavirus Disease 2019 Pandemic on Their Occurrence. *Clin Infect Dis* .2023;77(Suppl 1) : 10.1093/cid/ciad260 S29-S37 10.1093/cid/ciad260 37406041 PMC10321697

[5] : Increased Detection of Carbapenemase-Producing Enterobacterales Bacteria in Latin America and the Caribbean during the COVID-19 Pandemic. *Emerg Infect Dis* .2022;28(11) : 10.3201/eid2811.220415 1-8 10.3201/eid2811.220415 36286547 PMC9622262

# Reviewer report: Reviewer response for version 1

**DOI:** 10.21956/wellcomeopenres.25914.r117208

**Published:** 2025-02-05

**Authors:** Jens Thomsen

**Affiliations:** 1 Medics Labor AG, Microbiology, Bern, Switzerland; 2 Khalifa University, Abu Dhabi, Abu Dhabi, United Arab Emirates

This manuscript describes and discusses the work of the SEDRIC consortium between 2018-2024, highlighting successes and milestones, and providing examples where technical analyses and recommendations have helped to inform policy and funding priorities to improve the surveillance and epidemiology of drug-resistant infections. 

 The manuscript is very well written, by experts in the field, is very concise and highly informative, summarizing nicely the work and achievements of SEDRIC during that period. It touches on many important aspects to be considered for the successful surveillance of AMR and drug-resistant pathogens, with a special focus on LMICs, and the related challenges and limitations.

 All factual statements are correct, to my knowledge, and are adequately supported by citations and references.

 I would strongly recommend this open letter to  be indexed without further modification.

 Congratulations on this well written paper.

Does the article adequately reference differing views and opinions?

Yes

Are all factual statements correct, and are statements and arguments made adequately supported by citations?

Yes

Is the Open Letter written in accessible language?

Yes

Where applicable, are recommendations and next steps explained clearly for others to follow?

Yes

Is the rationale for the Open Letter provided in sufficient detail?

Yes

Reviewer Expertise:

Clinical Microbiology and Infectious Disease Epidemiology, AMR Surveillance, GLASS, WHONET, Infection Prevention and Control, Public Health

I confirm that I have read this submission and believe that I have an appropriate level of expertise to confirm that it is of an acceptable scientific standard.
